# Pre-calving Intravaginal Administration of Lactic Acid Bacteria Reduces Metritis Prevalence and Regulates Blood Neutrophil Gene Expression After Calving in Dairy Cattle

**DOI:** 10.3389/fvets.2018.00135

**Published:** 2018-06-21

**Authors:** Sandra Genís, Ronaldo L. A. Cerri, Àlex Bach, Bruna F. Silper, Matheus Baylão, José Denis-Robichaud, Anna Arís

**Affiliations:** ^1^Department of Ruminant Production, Institut de Recerca i Tecnologia Agroalimentàries, Barcelona, Spain; ^2^Applied Animal Biology, Faculty of Land and Food Systems, University of British Columbia, Vancouver, BC, Canada; ^3^Institució Catalana de Recerca i Estudis Avançats, Barcelona, Spain; ^4^Department of Population Medicine, Ontario Veterinary College, University of Guelph, Guelph, ON, Canada

**Keywords:** intra-vaginal, intra-uterine, lactic acid bacteria, metritis, neutrophil

## Abstract

Metritis affects up to 40% of dairy cows and it is usually treated with antibiotics. In spite of their advantages, there is an increased concern about antibiotic resistance leading to the research of alternative methods. The aim of this study was to evaluate the effects of a combination of lactic acid bacteria (LAB) on the prevalence of metritis and modulation of endometrial and neutrophil inflammatory markers in dairy cows. One hundred and thirty-five cows were enrolled 3 week before calving and randomly assigned to three treatments. Treatment groups were: (1) two intravaginal doses of LAB/wk during 3 week pre-calving (vaginal, *n* = 45); (2) an intra-uterine dose, once 1 d after calving (uterine, *n* = 44); and (3) no intervention (CTRL, *n* = 45). Metritis was defined as body temperature > 39.5°C and purulent vaginal discharge (> 50% pus), and diagnosed 6 d after calving. Blood samples were taken at d −14, −10, −7, −4, +1, +3, +6, and +14 relative to calving for non-esterified fatty acids (NEFA) analysis. At d −10, +1, +3, and +6 neutrophils were isolated from blood for gene expression analysis by RT-qPCR. Endometrium biopsies were taken from 30 cows, 15 from CTRL and 15 from the uterine group at d +1, +3, and +6 after calving for pro-inflammatory markers analysis by NanoString®. Vaginal treatment reduced metritis prevalence (6/45) up to 58% compared with CTRL group (14/45), but there was no difference between the uterine and CTRL group. Uterine and vaginal treatments reduced blood neutrophil gene expression. Expression of pro-inflammatory markers in the endometrium did not differ between uterine and CTRL cows. Metritic cows expressed more C-X-C motif chemokine ligand 8 (*CXCL8*) and interleukin 1 beta (*IL1B)* at d 3 than healthy cows, whereas healthy cows expressed more *CXCL8* at d 1 relative to calving in the endometrium. This study shows a promising potential of LAB probiotics as a preventive treatment against metritis in dairy cows.

## Introduction

Acute metritis is an inflammation of the uterus due to bacterial infection occurring during the first 21 days after calving [with greater prevalence during the first 7 days in milk (DIM)] (1). The main characteristics are systemic signs of sickness, including fever over 39.5°C, red-brown watery foul-smelling vaginal discharge, dullness, inappetence, elevated heart rate, and low milk production (2). Damage to the uterine tissue during parturition leads to inflammation that likely contributes to the systemic condition (3). This inflammation in the uterus comes from the need for substantial tissue repair as part of post-partum involution characterized by the recruiting of immune cells (4). When microorganisms reach the uterus, the development of disease depends on the balance between the host defense systems, the virulence of the microbes, and the environmental bacterial load (5).

Traditional antimicrobial treatments against metritis are not very effective as only 67–77% of treated cows decrease fever after 5–10 days of treatment and there is not always resolution of the fetid odor (1). Furthermore, an improvement of the reproductive performance of the treated animals is not always found, especially in cases of sustained inflammation (1). The recommendations from the World Health Organization (6) to reduce antibiotic treatments leads to investigate alternative therapies.

The use of probiotic strains has been proposed as an alternative to prevent postpartum uterine infections and inflammation. A Canadian group (7) demonstrated that some lactic acid bacteria (LAB) strains were able to reduce uterine disease prevalence in dairy cows. Our recent work has also demonstrated that other LAB strains are able to modulate uterine inflammation and decrease *Escherichia coli* infection *in vitro* and *ex vivo* (8, 9). Interestingly, when LAB were administered intra-vaginally they altered the vaginal environment and tended to restrict the amount of bacteria reaching the uterus despite the fact that LAB did not reach the endometrium (10).

Periparturient dairy cows have nutritional requirements that exceed their dietary intake potential. This leads to a state of negative energy balance (NEB), during which body tissue reserves are mobilized to provide energy, hampering the resolution of peripartum diseases (11). Cows with a greater degree of NEB have higher concentrations of non-esterified fatty acids (NEFA) in blood, which are also associated with decreased feed intake (12, 13).

Parturition in dairy cattle is associated with impairment of neutrophil phagocytosis and oxidative burst activity (14), which leads to a decreased ability to fight bacterial infections. A study (11) demonstrated *in vitro* that an increase of NEFA in blood tended to desensitize neutrophil oxidative burst, which could compromise uterine health because specific phagocytosis by neutrophils is the most important component of uterine defense after bacterial infections (15). During inflammation, L-selectin (SELL), an adhesion molecule expressed on the neutrophil surface, promotes neutrophil infiltration into the infected tissue (16). When the neutrophils are in the infected tissue they recognize and bind pathogens and proceed to phagocytose and destroy them. One of the mechanisms they use is the respiratory burst which some of their components are the neutrophil cytosolic factor 1 (NCF1), a component of the NAPDH oxidase enzyme complex, and the superoxide dismutase (SOD) (17).

The idea behind this treatment was to study not only its effect on metritis prevalence but also to study the difference between an intra-uterine and intra-vaginal application and whether a more direct application of LAB to the endometrium would be able to down-regulate post-calving inflammation. In consequence, the aim of this study was to test two different approaches to apply a LAB combination in dairy cows (vaginal or uterine) to reduce metritis prevalence and to study the influence of LAB on NEFA concentrations, and gene expression of pro-inflammatory cytokines in blood neutrophils and endometrium.

## Materials and methods

### Ethics statement

This experiment was conducted in the facilities of the Dairy Research and Educational Centre from the University of British Columbia (UBC) in Agassiz, Canada. All experimental procedures were approved by the UBC Animal Care Committee.

### Animals and experimental design

One hundred and thirty-five cows (45 primiparous and 90 multiparous) were enrolled 3 week before calving and randomly assigned to treatments using frequency matching to ensure similar frequencies for parity and previous illness in all treatment groups. The treatment groups were: (1) two intravaginal doses of lactic acid bacteria (LAB) per week during 3 week pre-calving (vaginal, *n* = 45); (2) one intra-uterine dose 1 day after calving (uterine, *n* = 45); and (3) no intervention (CTRL, *n* = 45). The average number of doses that the cows belonging to the vaginal treatment received was 5. The LAB treatment dose was a mixture of *Lactobacillus rhamnosus* CECT 278 (Colección Española de Cultivos Tipo, Valencia, Spain), *Pediococcus acidilactici* CECT 5,915, and *Lactobacillus reuteri* DSM 20016 (German collection of microorganisms and cell cultures, Leibniz, Germany) with a final cell count of 4.5 × 10^10^ CFU/dose and a proportion of 25/25/2, respectively. The LAB treatment was made fresh every day. Intravaginal LAB treatments were applied with a plastic cannula, previously cleaned with water and 70% ethanol, placed in the vagina just before the cervix. For the uterine treatment, 12 mL of LAB was applied with a sterile syringe directly inside the uterus after cleaning the exterior of the vagina with iodine. Aseptic procedures were maintained during LAB administration. A small sample from the endometrium was collected through a non-surgical procedure (1, 3, and 6 d after calving) from 30 cows randomly chosen (15 cows from the uterine treatment and 15 cows from the CTRL group). No cows from the vaginal treatment were biopsied because it had been previously shown that LAB treatment applied in the vagina does not reach the endometrium and does not modulate gene expression of pro-inflammatory cytokines (10). An epidural anesthesia was done using 100 mg of lidocaine (Lidocaine HCl 2%, Vetoquinol, Lavaltrie, QC, Canada). The vulva was cleaned, and a disinfected guarded biopsy instrument (crocodile-type biopsy forceps, made by Aries Surgical, Davis, CA, USA) was introduced via the cervix in the body of the uterus by vaginal (d 1) or trans-rectal (d 3, and 6) manipulations. Tissue collected was submerged in 500 μL of RNAlater (Thermo Fiser Scientific, Cramlington, UK) and kept overnight at 4°C. Then RNAlater was removed and the tissue stored at −80°C until further analysis.

### Clinical observations and measurements

All cows were monitored clinically at 1, 3, and 6 DIM for metritis. Rectal temperature was measured twice and fever was defined as a temperature greater than 39.5°C. Retained placenta was diagnosed if a cow did not expel the placenta within 24 h after parturition. Cows with retained placenta were removed from the study as they were treated with antibiotics following the farm's standard procedures. A case of metritis was defined as a cow with reddish brown vaginal discharge, fetid odor, at least 50% pus and fever at 6 DIM. The Metricheck device, a soft rubber hemisphere connected to a stainless steel rod, was inserted into the vagina to assess the vaginal discharge. Vaginal discharge was evaluated after retracting the device caudally (18).

### Sampling and laboratory analysis

Blood samples were collected from the coccygeal vein into 10 mL vacutainer tubes with ethylenediaminetetraacetic acid (EDTA, BD Vacutainer Systems, Plymuth, UK) on −14, −10, −7, −4, +1, +3, +6, and +14 d relative to calving. Blood samples were processed for neutrophil RNA isolation or centrifuged at 1,573 × *g* at 5°C for 15 min to separate plasma. Two aliquots were stored at −20°C until further analysis. Concentrations of NEFA were measured in plasma of d −10, +1, +3, and +6 using a commercially available kit (Wako Diagnostics, Mountain View, USA) within 2 months after sampling.

### Bacterial strains and culture conditions

Bacterial cultures were grown inoculating 1 mL of a glycerinate in 45 mL of Man, Rogosa, and Sharpe medium (MRS, Scharlau, Sentmenat, Spain) for *L. rhamnosus*, and *P. acidilactici*; and in 20 mL of MRS medium for *L. reuteri* at 37°C in static conditions for 2 days. Then bacteria were centrifuged at 6,000 × *g* for 15 min and re-suspended with sterile NaCl with a final concentration of 4.5 × 10^10^ CFU/dose. Each dose had a ratio of 25/25/2 for *L. rhamnosus, P. acidilactici*, and *L. reuteri*, respectively.

### Neutrophil isolation

Neutrophils were isolated from 20 mL of blood based on the procedures described by Carlson and Kaneko (19) and Farinacci et al. (20) from blood extracted at 10 d before calving, and at d +1, +3, +6 after calving. Specifically, blood was centrifuged at 1,000 × *g* for 45 min at 4°C, and then plasma, buffy coat, and two-thirds of the red blood pack were removed. Red blood cells in the remaining sample were lysed by adding 12 mL of hypotonic solution (10.6 mM Na_2_HPO_4_, and 2.7 mM NaH_2_PO_4_) and mixing for 90 s. Then, 6 mL of 4°C hypertonic solution (10.6 mM Na_2_HPO_4_, 2.7 mM Na_2_PO_4_, and 430 mM NaCl) were added, and tubes were mixed to restore isotonicity. Samples were centrifuged at 800 × *g* for 5 min at 4°C. The supernatant was discarded and the pellet was washed with 10 mL of Hank's Balanced Salt Solution (HBSS, Sigma-Aldrich, St. Louis, USA). Then samples were centrifuged again at 800 × *g* for 5 min at 4°C and the pellet was washed with 10 mL of HBSS. Samples were centrifuged at the same conditions and the pellet resuspended with 1 mL of HBSS and poured into 1.5 mL microcentrifuge tubes. The tubes were centrifuged at 9,600 × *g* for 15 min at 4°C, the supernatant was pipetted off, and 500 μL of TRIzol reagent (5Prima, Gaithesburg, USA) was added. Samples were stored at −20°C until RNA extraction.

### Reverse transcription and quantitative PCR (RT-qPCR)

Total RNA was extracted from neutrophil isolations using TRIzol Reagent (5Prima, Gaithesburg, USA) following manufacturer's instructions and quantified using a Nanodrop instrument (Thermo Fisher Scientific, Cramlington, UK). The RNA was reverse transcribed to DNA using iScript cDna Synthesis kit (Bio-Rad, CA, USA) using 2 μL with a concentration of 50 ng/μL. Reactions of RT-qPCR were performed using CFX384 Touch Real-Time PCR Detection System (Bio-Rad, CA, USA) and RT-qPCR conditions for each set of primers were optimized (Table 1). The specificity of the amplification was evaluated by the single band identification at the expected molecular weight in DNA agarose gel and a single peak in the RT-qPCR melting curves. The efficiency was calculated by amplifying serial 1/10 dilutions of each gene amplicon. A standard curve at Ct vs. log was plotted to obtain the efficiency, which was calculated using the formula 10^1/slope^, with an acceptable range of 1.8–2.05 (21). A total reaction volume of 20 μL was used, 10 μL of SYBER Green Fluoresecent (Bio-rad, California, USA), and the optimal primer concentration for each gene (Table 1). The RT-qPCR reactions were cycled as follows: an initial denaturalizing step of 10 min at 95°C, followed by 40 cycles of 10 s at 95°C, 15 s at optimized annealing temperature for each gene, 30 s at 72°C and a final extension of 10 min at 72°C. Relative gene expression was calculated using the Pfaffl method (22) with glyceraldehyde-3-phosphate dehydrogenase (*GAPDH)* and ribosomal protein S9 (*RPS9*) as reference gene controls. All RT-qPCR reactions were performed in triplicate. *GAPDH* and *RPS9* were chosen because they are stably-expressed in a variety of cells, including uterine tissues (23, 24).

**Table 1:** Sequence, annealing temperatures (At), concentration (μM), amplicon size (bp) of forward (Fw), and reverse (Rv) bovine primers used for RT-qPCR and accession number for neutrophil gene expression (17).

**Gene**	**Fw**	**Rv**	**At, C°**	**μm**	**bp**	**Accession number**
*GAPDH^*a*^*	GCATCGTGGAGGGACTTATGA	GGGCCATCCACAGTCTTCTG	52	0.125	67	NM_001034034.2
*RPS9^*b*^*	CCTCGACCAAGAGCTGAAG	CCTCCAGACCTCACGTTTGTTC	57	0.125	63	NM_001101152.2
*SELL^*c*^*	ACGGGAAAAAAGGATTACTATGGA	GCCTATAGTTGCATATGTATCAAATTTTCA	51	0.25	144	NM_174182.1
*NCF1^*d*^*	TCCTCAACTTCTTCAAGGTGCG	CAGCGTTGTTCTTGCCATCTTT	53	0.5	107	NM_174119.4
*SOD1^*e*^*	GGCTGTACCAGTGCAGGTCC	GCTGTCACATTGCCCAGGT	55.8	0.25	100	NM_174615.2
*TNFaR^*f*^*	GTGCAGTGCGTGTGTTTGTGTC	ATCTTCGCAACCACTGCCTTG	55.2	0.5	109	NM_174674.2

a *Glyceraldehyde-3-phosphate dehydrogenase*.

b *Ribosomal protein S9*.

c *Selectin L*.

d *Neutrophil cytosolic factor 1*.

e *Superoxide dismutase 1*.

f *Tumor necrosis factor-alpha receptor*.

### Nanostring® nCounter assay

Gene expression from biopsy samples was measured on the NanoString® nCounter Analysis System (NanoString® Technologies, Washington, USA). The system measures the relative abundance of each mRNA transcript of interest using a multiplex hybridization assay as digital readouts of fluorescent barcode probes that are hybridized to each transcript. An nCounter CodeSet (NanoString® Technologies, Washington, USA) containing a biotinylated capture prove for 4 genes and four reference genes (Table S1), and reporter proves attached to a color barcode tags according to the nCounter™ code-set design was hybridized in solution to 200 ng of total RNA for 18 h at 67°C according to the manufacturer's instructions.

### Bioinformatics

To obtain gene expression data from the NanoString® nCounter assay, filtering of samples using quality control (QC) criteria was performed according to the manufacturer's recommendations. Row counts of QC-passed samples were normalized using four reference genes as internal controls: *GAPDH*, actin beta (*ACTB), RPL19*, and phosphoglycerate kinase 1 (*PGK1*).

### Statistical analysis of results

All data were analyzed using SAS 9.2 software (SAS Institute Inc., Cary, NC). The number of animals that needed to be enrolled was determined with a power analysis. Metritis prevalence rate in the experimental farm was 33%, thus based on that, we hypothesized that the vaginal and/or the uterine treatment would be able to reduce the incidence rate by 15% (half). A power analysis using a nominal power of 85% at a 0.05% two-sided significant level indicated a minimum sample size of 45 (45 cows in each group) to detect treatment differences. One hundred fifty-eight cows were enrolled into this study, 7 cows were discarded because of retained placenta, 5 due to abortion, and 12 were culled resulting in 134 cows included in the statistical analysis.

Prior to statistical analysis, data were either log- or square root-transformed when necessary to achieve a normal distribution of the residuals. *SELL, SOD1, NCF1, TNFaR, CXCL8, IL1B, IL6*, and *NEFA* data was log-transformed while *TNFa* data was square root-transformed. Results herein are reported as the means of non-transformed data ± SEM obtained with normalized data (except otherwise indicated). Neutrophils' gene expression, blood NEFA concentration, and NanoString® data were analyzed using an ANOVA, considering treatment, parity, and day as fixed effects, and animal as a random effect. Three-way interaction between treatment, metritis, and day were tested on the NanoString® data. Stratified analysis was performed to evaluate differences in gene expression between metritic and healthy cows with NanoString® results, thus removing all data from treated cows (uterine treatment) and only considering those belonging to the CTRL group. Interaction between neutrophil gene expression and NEFA concentration were tested. Differences were considered significant when *P* < 0.05. Metritis prevalence was analyzed using a Chi-square comparing each treatment against CTRL cows.

## Results

### Metritis prevalence

Cows in the vaginal treatment group (6/45, metritic cows/total) had a lower prevalence of metritis compared with cows belonging to the CTRL group (14/45, *P* = 0.04), whereas the uterine treatment (13/44) did not differ from the CTRL group (*P* = 0.87). Vaginal treatment lowered the prevalence of metritis by 58% compared with the CTRL group, resulting in a prevalence of 13.3%; whereas the CTRL group had a prevalence of metritis of 31.1%. Primiparous cows (16/44, metritic cows/total) had a greater (*P* = 0.02) metritis prevalence than multiparous cows (16/88).

### Neutrophil relative gene expression

**Figure 1:**
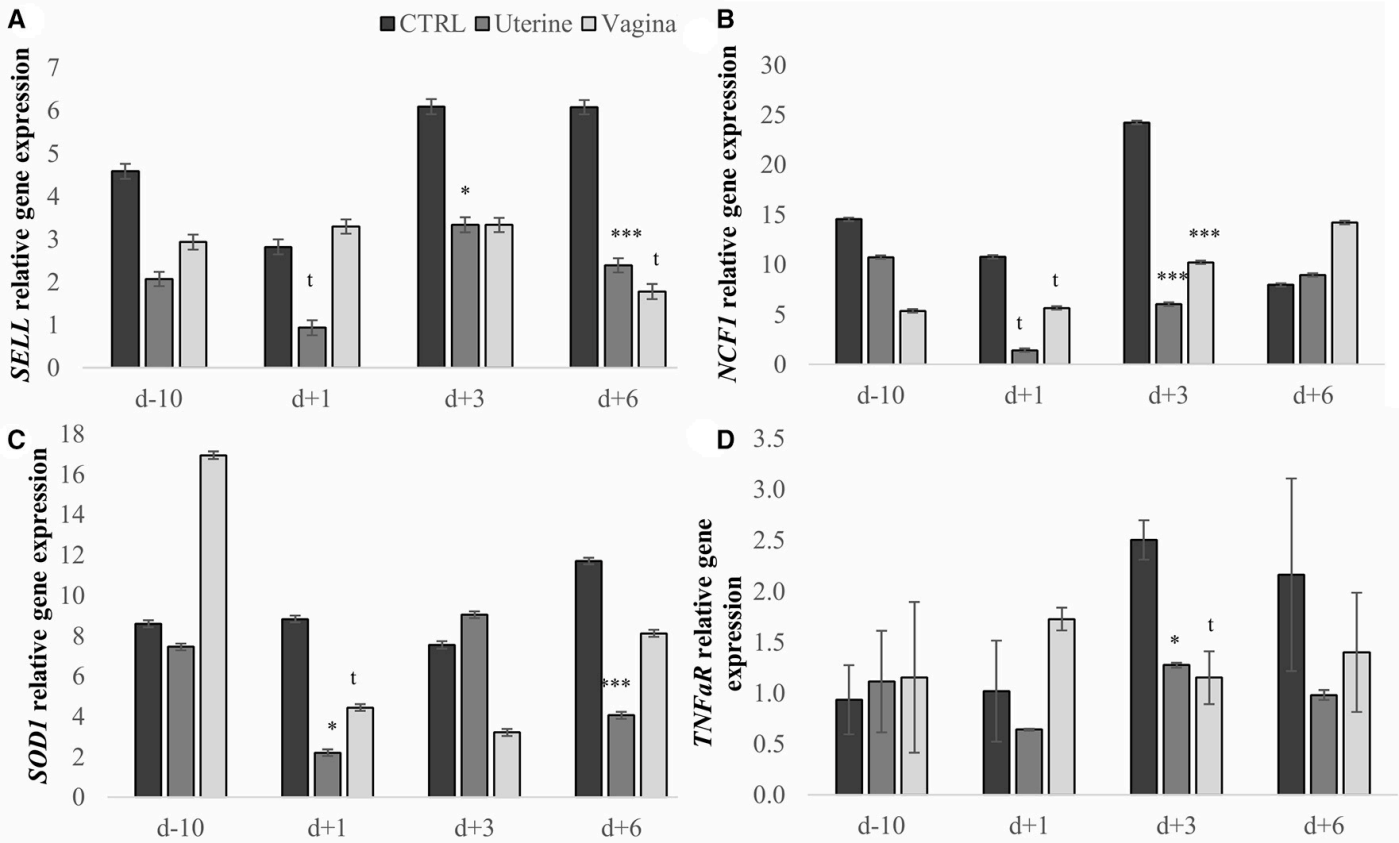
Neutrophil relative gene expression by treatment per day in dairy cows. Expression of *selectin L* (*SELL*, **A**), expression of *neutrophil cytosolic factor 1 (NCF1*, **B**), expression of *superoxide dismutase 1 (SOD1*, **C**), and expression of *tumor necrosis factor-alpha receptor (TNFaR*, **D**). CTRL, Control cows (untreated); Uterine, one intra-uterine dose of lactic acid bacteria (LAB) 1 day after calving; Vaginal, two intravaginal doses of LAB per week during 3 week pre-calving. Bars represent means ± SEM for different conditions. Bars with *t* (*P* < 0.1), *P < 0.05 or ***P < 0.005 differ from CTRL treatment. D-10, 10 days before calving; d1, day 1 after calving; d3, day 3 after calving; d6, day 6 after calving.

The expression of *SELL, NCF1, SOD1*, and *TNFaR* genes was used as a marker of neutrophil potential killing activity (17). Treatment affected the expression of *SELL, NCF1, SOD1*, and *TNFaR (P* = 0.002, *P* = 0.014, *P* = 0.014 and *P* = 0.035, respectively; Table 2). The uterine treatment reduced 2.26-fold *SELL*, 2.08-fold *NCF1*, 1.62-fold *SOD1*, and 1.69-fold *TNFaR* gene expression compared with the CTRL, whereas vaginal treatment reduced *SELL* and *TNFaR* gene expression (1.72-fold, 1.22-fold, respectively), and had a tendency to reduce *NCF1* (*P* = 0.056). When we analyzed the expression of each gene by treatment per day we observed that the uterine treatment tended to decrease *SELL* gene expression at day 1 and reduced it at day 3 and 6 compared with CTRL treatment (Figure 1A, *P* = 0.053, 0.035, and 0.007, respectively) and that the vaginal treatment tended to reduce *SELL* gene expression at day 6 (*P* = 0.092). *NCF1* gene expression tended to decrease with the uterine and the vaginal treatments at day 1 (*P* = 0.057 and 0.098, respectively), were downregulated at day 3 (*P* = 0.007 and 0.004, respectively), and again, there was a tendency to decrease *NCF1* expression at day 6 compared with CTRL treatment (Figure 1B, *P* = 0.093). The expression of *SOD1* was reduced in the uterine treatment at days 1, and 6 and tended to be decreased at day 3 compared with CTRL and also compared with the vaginal treatment at day 6 (Figure 1C, *P* = 0.028, 0.096, 0.004, and 0.028, respectively). Uterine treatment reduced and the vaginal treatment tended to decrease the expression of *TNFaR* gene compared with the CTRL treatment (Figure 1D, *P* = 0.013 and 0.088, respectively).

**Table 2:** Neutrophil gene expression by treatment on dairy cows (relative units of gene expression).

		**Treatment^1^**			
**Gene**	**CTRL**	**Uterine**	**Vaginal**	**SEM**	***P*-value**
*SELL*^2^	4.92^a^	2.18^b^	2.86^b^	0.092	0.002
*NCF1*^3^	14.21^a^	6.81^b^	9.05^a,b^	0.119	0.014
*SOD1*^4^	9.27^a^	5.72^b^	7.83^a^	0.097	0.014
*TNFαR*^5^	1.67^a^	0.99^b^	1.37^b^	0.088	0.035

1 *CTRL, Control cows (untreated); Uterine, one intra-uterine dose of lactic acid bacteria (LAB) 1 d after calving; Vaginal, two intravaginal doses of LAB per week during 3 week pre-calving*.

2 *Selectin L*.

3 *Neutrophil cytosolic factor 1*.

4 *Superoxide dismutase 1*.

5 *Tumor necrosis factor-alpha receptor*.

a, b *Means within a row with different subscripts differ (P < 0.05)*.

Metritis also affected the expression of *TNFaR* (*P* = 0.041, Table 3). Cows with metritis expressed 1.65-fold more *TNFaR*, and tended to express more *SELL* (*P* = 0.079) than healthy cows. Furthermore, day of sampling tended to affect the expression of *NCF1* (*P* = 0.070, data not shown), because *NCF1* expression was down-regulated at day 3 compared with day 1. No interaction was significant between treatment and metritis (*SELL P* = 0.548, *SOD1 P* = 0.298, *NCF1 P* = 0.651, and *TNFaR P* = 0.343), or day and metritis for the evaluated genes.

**Table 3:** Neutrophil gene expression as affected by presence or absence of metritis in dairy cows.

	**Metritis**			
**Gene**	**No**	**Yes**	**SEM**	***P*-value**
*SELL*^1^	2.60^a^	4.02^a^	0.075	0.080
*NCF1*^2^	10.01^a^	9.97^a^	1.812	0.329
*SOD1*^3^	5.69^a^	9.67^a^	0.080	0.374
*TNFαR*^4^	1.01^b^	1.67^a^	0.072	0.041

a, b * Means within a row with different subscripts differ (P < 0.05)*.

1 *Selectin L*.

2 *Neutrophil cytosolic factor 1*.

3 *Superoxide dismutase 1*.

4 *Tumor necrosis factor-alpha receptor*.

### Non-esterified fatty acids (NEFA)

**Figure 2:**
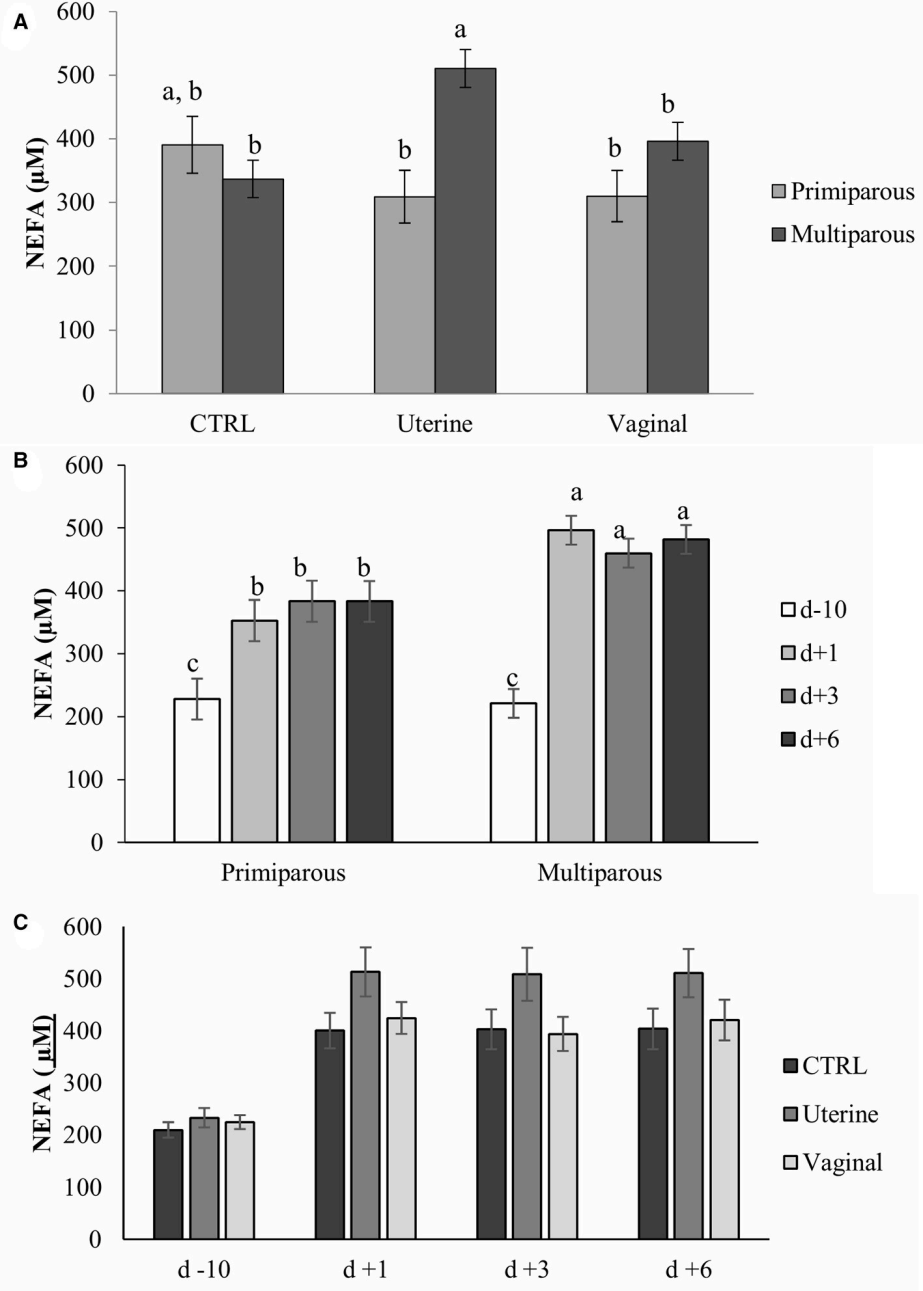
Blood non-esterified fatty acids (NEFA) concentrations by treatment and parity **(A)**, by parity and day **(B)**, and by treatment and parity **(C)** in dairy cows. Bars represent mean ± SE. Bars with different letters differ (*P* < 0.05). CTRL, Control cows (untreated); Uterine, one intra-uterine dose of lactic acid bacteria (LAB) 1 day after calving; Vaginal, two intravaginal doses of LAB per week during 3 week pre-calving; D-10, 10 days before calving; d1, day 1 after calving; d3, day 3 after calving; d6, day 6 after calving.

Blood NEFA concentration was associated with day (*P* < 0.01), and parity (*P* < 0.05), but not with treatment (*P* = 0.45). Interactions between treatment and parity (*P* < 0.01; Figure 2A), and parity and day (*P* < 0.01; Figure 2B) were significant; but no interaction between treatment and day (*P* = 0.48, Figure 2C) was observed. Blood NEFA concentration was higher in multiparous cows of the uterine group than in primiparous cows of both the uterine treatment and multiparous cows of CTRL group (Figure 2A). Blood NEFA concentration was 112.67 μM less before calving (d −10) than after calving (d +1, +3, +6) in primiparous cows, and was 239.57 μM higher after calving (d +1, +3, +6) than before calving (d −10) in multiparous cows. No differences were observed between heifers and cows before calving, but after calving, multiparous cows had at least 1.20-fold higher NEFA concentration in blood than primiparous cows. No differences in NEFA concentrations were found between metritic and healthy cows (data not shown). There were low or no correlations between neutrophil gene expression (*SELL, NCF1, SOD1*, and *TNFaR*) and blood NEFA concentrations. The *r*^2^ were 0.02, 0.04, 0.02, and 0.01 respectively with *P*- values of 0.10, 0.03, 019, and 0.32.

### Pro-inflammatory markers

The expression of genes coding for the pro-inflammatory cytokines C-X-C motif chemokine ligand 8 (*CXCL8*), interleukine 1 beta (*IL1B)*, interleukin 6 (*IL6)*, and tumor necrosis factor-alpha (*TNFa)* was evaluated as markers of inflammation in the endometrium. The gene expression of these cytokines was affected by time (*P* < 0.01), but not by treatment or metritis. No interactions were found between treatment and metritis, metritis and time, time and treatment. Nor was the triple interaction among treatment, metritis, and time.

**Figure 3:**
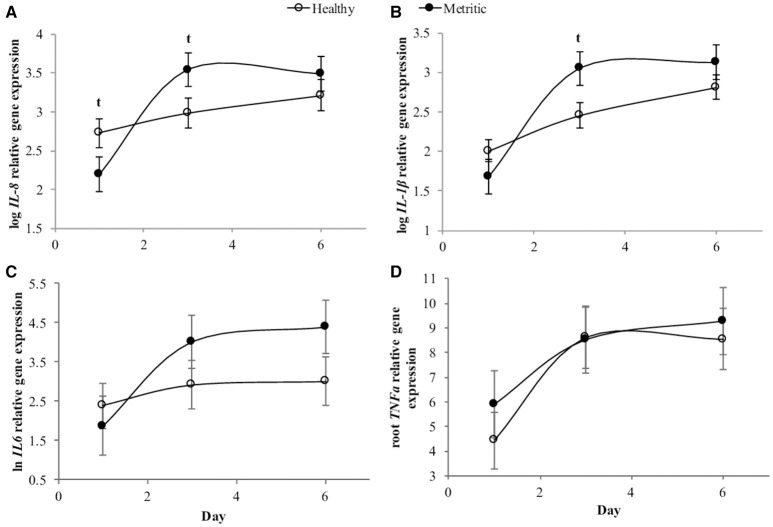
Cytokine gene expression from endometrial biopsies taken from healthy (open circles) and metritic dairy cows (solid circles) from the CTRL treatment. Expression of C-X-C motif chemokine ligand 8 *(CXCL8)* gene over time **(A)**, interleukine 1 beta (*IL1B*, **B**), interleukin 6 *(IL6*, **C**), and tumor necrosis factor- alpha (*TNFa*, **D**). Points represent means ± SEM for different conditions. Points with *t* differ (*P* < 0.1) between healthy and metritic cows.

As treatment did not affect the expression of pro-inflammatory profile, a second analysis was performed to evaluate differences in gene expression between 6 metritic and 6 healthy cows, only using cows in the CTRL group. The gene expression of *CXCL8* was affected by day (*P* < 0.01), and there was an interaction between metritis and day (*P* < 0.05; Figure 3A). Healthy cows tended to express more *CXCL8* at day 1 (*P* = 0.08, *2*.30-fold) whereas metritic cows tended to express *CXCL8* (*P* = 0.07) by 2.45-fold at day 3 compared with healthy cows. The gene expression of *IL1B* was affected by day (*P* < 0.01) and there was an interaction between day and metritis (*P* < 0.05; Figure 3B). Metritic cows tended (*P* = 0.053) to have a 3.05-fold greater expression of *IL1B* gene at day 3 compared with healthy cows. No differences were found at days 1 and 6. The expression of *IL6* and *TNFa* was affected by day (*P* < 0.05), but no differences were observed between healthy and metritic cows (Figures 3C,D).

## Discussion

The main objective of this study was to evaluate the capacity of a combination of LAB strains which previously showed to reduce *E. coli* infection *in vitro* and *ex vivo* (8), to modulate inflammation and reduce the prevalence of metritis after intra-vaginal or intra-uterine administration. Previous work by other authors showed that intra-vaginal administration of other LAB strains was able to reduce uterine infections in dairy cows (7). Most of the probiotics currently used in animals are LAB, especially in the case that they are used to try to modulate infection and/or inflammation. In our study, the intra-vaginal application of LAB twice per week during the 3 week before calving was able to reduce the prevalence of metritis compared with the CTRL cows. Recent studies had characterized the uterine microbiota from cows (25, 26) showing a different microbial community from metritic or healthy cows. On the first study (27) they found that *Fusobacteria* was the dominant group in metritic cows, while *Gamaproteobacteria* was more present in the uterus of healthy cows. The second study (28) also observed an increase abundance of *Fusobacteria* in metritic cows along with and increment of *Bacteroidetes*. Even then, *E. coli* is still being described as the main pathogen responsible for initiating uterine infection and inflammation, unbalancing the uterine microbiota and favoring the infection of other pathogens such as *Bacteroides* spp. or *Fusobacterium necrophorum* (28). Also, it has been previously shown that cows treated intra-vaginally with this combination of LAB tended to have lesser presence of *E. coli* in the vagina than not-treated cows (*P* = 0.09) (10). These findings could explain partially the results observed herein.

On the other hand, the intra-uterine application of the same dose of LAB did not show any difference (*P* = 0.872) in metritis prevalence compared to the CTRL group.

In this study, neutrophil gene expression was quantified as part of the inflammatory response. It is known that neutrophils, are a primary source of immune response in the uterus, with the neutrophil activity being associated with risk of metritis (12). Some authors (29) have stated that a desirable response should be a prompt, substantial flux of neutrophils into the uterus after calving. On the other hand, an excessive immune response could be counter-productive as it impairs uterine or ovarian function (15). In the current study metritic cows tended (*P* = 0.079; Table 2) to express more *SELL* (l-selectin), a protein that is expressed on the neutrophil surface and promotes neutrophil infiltration into the infected tissue, compared with healthy cows. Furthermore, metritic cows expressed more *TNFaR* [the TNF-α receptor that is involved in regulating neutrophil functions such as degranulation, release of oxygen metabolites, phagocytosis and adhesion to endothelium (30)] than healthy cows (*P* = 0.041; Table 3). In LAB-treated cows, the vaginal and uterine treatments down-regulated the expression of all genes studied as markers of neutrophil activity compared with CTRL cows (Table 2). Specifically, the expression of *SELL* and *TNFaR* was reduced along with the expression of *NCF1* and *SOD*, both involved in the respiratory burst. In the vaginal treatment, the decrease of neutrophil activity could probably be related to a reduction of pathogenic bacteria in the vagina and a consequent reduction in the incidence of infection in the uterus. There were no differences between CTRL and uterine cows in the expression of *CXCL8* nor with the other cytokines *IL1B, IL6*, and *TNFa*. This was an unexpected result because previous *in vitro* and *ex vivo* studies indicated that a direct treatment of the endometrium with LAB, modulated the levels of these cytokines (9). However, the uterine treatment was associated with a reduction in neutrophil gene expression; but probably the dose was not sufficient to modulate the expression of proinflammatory cytokines, or the metritis prevalence.

The differences between a pathological and physiological inflammation is not well understood and depends on the severity, timing and duration of inflammation (15). It is widely accepted that all early post-partum cows are in a pro-inflammatory state (3) and that damage to uterine tissue during calving leads to inflammation that likely contributes to the systemic condition. In this study, when the profile of pro-inflammatory cytokines was analyzed only in CTRL animals the results showed that metritic cows had impaired expression of *CXCL8* at day 1 compared with healthy cows, but at day 3 this situation reversed, with *CXCL8* and *IL1B* being over-expressed in metritic compared with healthy cows (Figure 3). It seems that cows with a less active immune system are more susceptible to bacterial infections [which is present in almost all dairy cows during the 2–3 week after calving (31)] ultimately resulting in metritis, as the immune systems reacts more slowly.

A previous study Hammon et al. (12) found that cows with puerperal metritis had greater plasma NEFA levels pre-partum than healthy cows. In the current study, no differences in blood NEFA concentration between healthy and metritic cows were observed (*P* = 0.183). The inconsistent results may be due to different timeline used to diagnose metritis. In the present study, metritis was diagnosed when temperature rose > 39.5°C along with the presence of vaginal purulent discharge at the 1st week postpartum, whereas others (12) classified cows as metritic if purulent discharge was observed between day 0 and 14 after parturition, regardless of presence of fever.

In the current study, multiparous cows receiving the uterine treatment had an increase of blood NEFA concentrations, which indicates a larger NEB in this group of cows compared with multiparous cows from the CTRL and vaginal groups. However, differences in NEB could not be attributed to changes in body condition or milk production since no differences in milk yield at 60 DIM (*P* = 0.55; data not shown) and BW between d −10 to d +21 related to calving were observed between treatments (*P* = 0.96; data not shown). Thus, as cows in the uterine treatment modified neutrophil gene expression (Table 2) that may indicate that LAB is affecting the energy metabolism either directly or indirectly consequence of a potentially greater energy consumption from the immune system (32, 33). It has been previously reported that high blood NEFA concentration is associated with impaired blood neutrophil functions (11, 12). Herein, we have not found any correlation between NEFA and the expression of some genes related to neutrophil activity, but we cannot discard other relationships with total neutrophil activity, not measured in the current study. It could be speculated that with an impaired neutrophil activity, the endometrium is recruiting more immune cells to sustain the involution processes, increasing the NEB of the animal, but this hypothesis could not be confirmed because total immune cell counts were not performed in this study neither activity assays.

## Conclusions

The vaginal application of the combination of lactic acid bacteria used herein reduced metritis prevalence in dairy cows compared with untreated animals; whereas, the prevalence of metritis was the same between untreated cows and cows treated with LAB intra-uterine. The gene expression of pro-inflammatory cytokines was down-regulated in circulating neutrophils from intra-vaginal and intrauterine treatments with lactic-acid bacteria, but no change was observed in the post-partum endometrium. The combination of lactic acid bacteria administration prior to calving appears to be a promising strategy to prevent uterine infections.

## Author contributions

SG, AA, and RC: Design of the study; SG and MB: LAB preparation; SG, BS, JD-R, and MB: Collection of health data and blood; BS and JD-R: Collection of endometrial biopsies; SG: Laboratory analysis and statistical analysis; SG, AA, RC, and ÀB: Interpretation of the data and drafting of the manuscript. All authors read and approved the final manuscript.

### Conflict of interest statement

The authors declare that the research was conducted in the absence of any commercial or financial relationships that could be construed as a potential conflict of interest.

## Abbreviations

LAB: lactic acid bacteria.
