# Supplementary material for: Pre-calving Intravaginal Administration of Lactic Acid Bacteria Reduces Metritis Prevalence and Regulates Blood Neutrophil Gene Expression After Calving in Dairy Cattle
Source: Front Vet Sci. 2018 Jun 21;5:135. doi: 10.3389/fvets.2018.00135 (PMC6021520; doi:10.3389/fvets.2018.00135)
Supplement: Supplementary file 1 [file Table_1.DOCX]

**Pre-calving intravaginal treatment of lactic acid bacteria reduces metritis prevalence and regulates blood neutrophil gene expression after calving in dairy cattle.**

Sandra Genís^1^, Ronaldo L. A. Cerri^2^, Àlex Bach^1, 3^, Bruna F. Silper^2^, Matheus Baylão^2^, José Denis-Robichaud^4^, Anna Arís^1, *^.

| Gene | Accession Number | Position | Target sequence |
| --- | --- | --- | --- |
| *ACTB* | NM_173979.3 | 56-155 | CGCCTTCGCCGCCGGTCGACACCGCAACCAGTTCGCCATGGATGATGATATTGCTGCGCTCGTGGTCGACAACGGCTCCGGCATGTGCAAGGCCGGCTTC |
| *GAPDH* | NM_001034034.1 | 213-312 | TGATTCCACCCACGGCAAGTTCAACGGCACAGTCAAGGCAGAGAACGGGAAGCTCGTCATCAATGGAAAGGCCATCACCATCTTCCAGGAGCGAGATCCT |
| *RPL19* | NM_001040516.1 | 193-292 | GCCTGTGACTGTCCATTCCCGGGCTCGATGCCGGAAAAACACCTTGGCTCGCCGGAAAGGCAGGCATATGGGTATAGGTAAGCGAAAGGGTACTGCCAAT |
| *PGK1* | NM_001034299.1 | 317-416 | CTGATGGTGTCCCCATGCCTGATAAGTACTCCTTGCAGCCAGTTGCTGTAGAACTCAAATCTCTGCTGGGCAAGGATGTTTTGTTCTTGAAGGACTGTGT |
| *CXCL8* | NM_173925.2 | 40-139 | CAGAAGAAACCTGACAAAAAGCCTCTTGTTCAATATGACTTCCAAGCTGGCTGTTGCTCTCTTGGCAGCTTTCCTGCTCTCTGCAGCTCTGTGTGAAGCT |
| *IL1B* | NM_174093.1 | 331-430 | TGACCTGAGGAGCATCCTTTCATTCATCTTTGAAGAAGAGCCTGTCATCTTCGAAACGTCCTCCGACGAGTTTCTGTGTGACGCACCCGTGCAGTCAATA |
| *IL6* | NM_173923.2 | 320-419 | CAAAAATGGAGGAAAAGGACGGATGCTTCCAATCTGGGTTCAATCAGGCGATTTGCTTGATCAGAACCACTGCTGGTCTTCTGGAGTATCAGATATACCT |
| *TNFa* | NM_173966.2 | 1207-1306 | TTCGCAACATTCCTTGAGAAGATCTCACCTAGAACTTGACATGCGTGGACTTCAACTCTCCCTTCCTGCCAATGTTTCCAGACTCCCCTGAGGTGGGAAG |

**Table S1:** *Accession number, position and target sequence for bovine genes* amplified with NanoString®.
